# Twisting and swiveling domain motions in Cas9 to recognize target DNA duplexes, make double-strand breaks, and release cleaved duplexes

**DOI:** 10.3389/fmolb.2022.1072733

**Published:** 2023-01-09

**Authors:** Jimin Wang, Pablo R. Arantes, Mohd Ahsan, Souvik Sinha, Gregory W. Kyro, Federica Maschietto, Brandon Allen, Erin Skeens, George P. Lisi, Victor S. Batista, Giulia Palermo

**Affiliations:** ^1^ Department of Molecular Biophysics and Biochemistry, Yale University, New Haven, CT, United States; ^2^ Department of Bioengineering and Department of Chemistry, University of California, Riverside, Riverside, CA, United States; ^3^ Department of Chemistry, Yale University, New Haven, CT, United States; ^4^ Department of Molecular and Cell Biology and Biochemistry, Brown University, Providence, RI, United States

**Keywords:** twisting motions, swiveling motions, open-closing motions, inactive-to-active transition, active site transformation, cleavage-ligation equilibrium, allostery

## Abstract

The CRISPR-associated protein 9 (Cas9) has been engineered as a precise gene editing tool to make double-strand breaks. CRISPR-associated protein 9 binds the folded guide RNA (gRNA) that serves as a binding scaffold to guide it to the target DNA duplex *via* a RecA-like strand-displacement mechanism but without ATP binding or hydrolysis. The target search begins with the protospacer adjacent motif or PAM-interacting domain, recognizing it at the major groove of the duplex and melting its downstream duplex where an RNA-DNA heteroduplex is formed at nanomolar affinity. The rate-limiting step is the formation of an R-loop structure where the HNH domain inserts between the target heteroduplex and the displaced non-target DNA strand. Once the R-loop structure is formed, the non-target strand is rapidly cleaved by RuvC and ejected from the active site. This event is immediately followed by cleavage of the target DNA strand by the HNH domain and product release. Within CRISPR-associated protein 9, the HNH domain is inserted into the RuvC domain near the RuvC active site *via* two linker loops that provide allosteric communication between the two active sites. Due to the high flexibility of these loops and active sites, biophysical techniques have been instrumental in characterizing the dynamics and mechanism of the CRISPR-associated protein 9 nucleases, aiding structural studies in the visualization of the complete active sites and relevant linker structures. Here, we review biochemical, structural, and biophysical studies on the underlying mechanism with emphasis on how CRISPR-associated protein 9 selects the target DNA duplex and rejects non-target sequences.

## Introduction

Bacteria have acquired innate immunity by incorporating palindromic DNA sequences into their own genome from invading viruses, phages, or plasmids. The function of the CRISPR (or Clustered Regularly Interspaced Short Palindromic Repeat) is to recognize and destroy invading phages or plasmids during reinfection. Cas9 (CRISPR-associated protein 9, particularly, from *Streptococcus pyrogenes* or SpyCas9) is one of the most extensively studied systems for which crystal and cryo-EM structures are known in many functional states (Doudna and Charpentier, 2014; Wang et al., 2016; Jiang and Doudna, 2017; Nidhi et al., 2021; Cofsky et al., 2022). Following structure determination, many biochemical and enzymological studies have been carried out using site-directed mutagenesis to define the catalytic sites of both the HNH and RuvC domains for cleavage of target and non-target DNA strands (tDNA and ntDNA). Other studies focused on kinetic pathways for recognition and selection of on-target DNA substrates for double-strand breaks. In this review, we examine recent literature on the molecular mechanisms of this enzyme, with emphasis on how the enzyme recognizes target DNA duplexes, makes double-strand breaks, and releases cleaved duplexes. Understanding such mechanisms is critical for the rational design of Cas9 enzymes with enhanced substrate selectivity in the context of gene editing tools.

**FIGURE 1:**
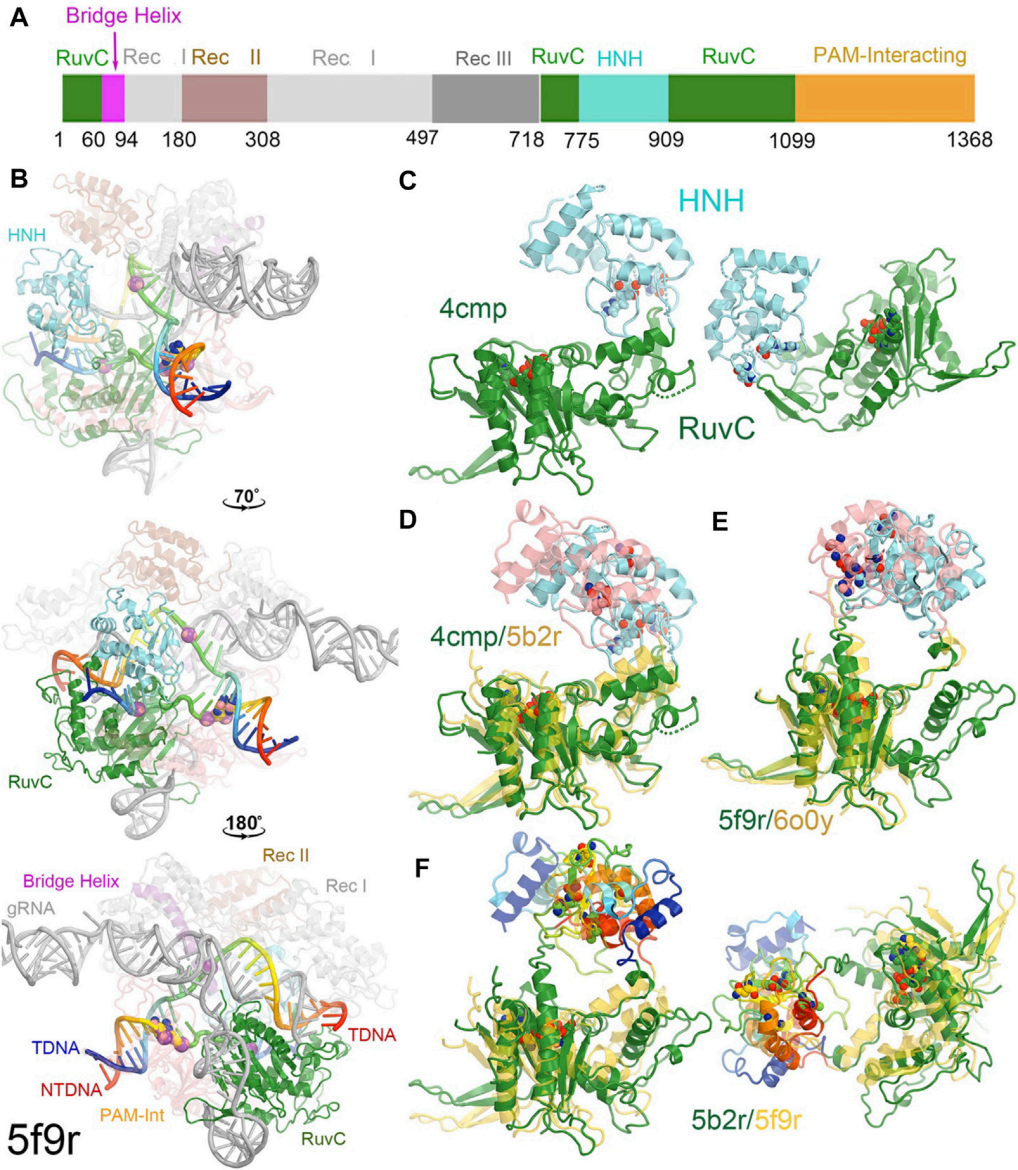
Overall structure of a RuvC-catalytically relevant Cas9 complex. **(A)** Linear structures with color coded domains. **(B)** Three different orientations of the 5f9r complex with successive rotations of about 70° and 180° along the vertical axis. Two strands of the DNA duplex are in rainbow colors, and gRNA is in grey. Two PAM nucleotides are in large balls-and-sticks. Scissile phosphates for both the tDNA and ntDNA strands are represented by large spheres. **(C)** Two views of the RuvC-HNH domains. **(D)** Superposition of the RuvC-HNH domains between the apo-4cmp and the catalytically inactive 5b2r structures. **(E)** Superposition of the catalytically relevant complexes of 5f9r and 6o0y. **(F)** Two views of the catalytically inactive and catalytically relevant complexes of 5b2r and 5f9r.

**FIGURE 2:**
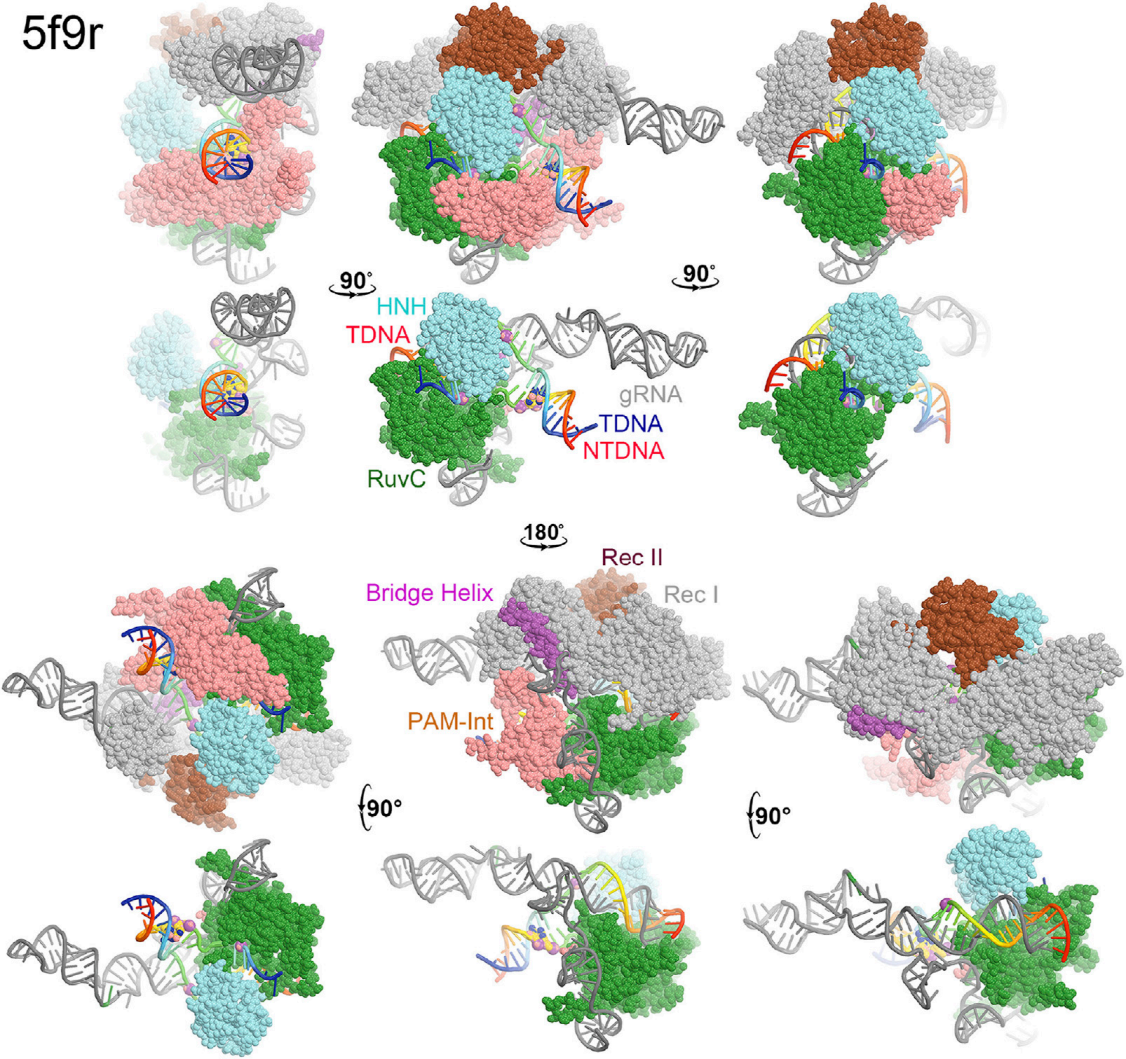
Surface representation of RuvC-catalytically relevant complexes in various orientations. Domains are colored as in Figure 1. Missing sidechains and loops were not rebuilt.

Due to the high flexibility of linkers and junctions of the protein domains, X-ray and Cryo-EM structures have encountered some difficulties in characterizing the activated enzyme, with studies reporting the visualization of the active sites only recently (Zhu et al., 2019; Bravo et al., 2022; Pacesa et al., 2022). For example, the overall resolution of the crystal structure reported for the RuvC-catalytically relevant complex (PDB accession number of 5f9r) is about 3.40 Å, and the global resolution of the cryo-EM map reported for the HNH-catalytically relevant complex (PDB accession ID, 6o0y/emd-0584) is 3.37 Å (Jiang et al., 2016; Zhu et al., 2019). With such limited resolution, many sidechains remained invisible in both nuclease catalytic sites and only poly(alanine) models could be built into the atomic models as in the reported coordinate files, which include the entire HNH poly(alanine) domain (and K548 and K510) in the 5f9r structures (Jiang et al., 2016). In many instances, even poly(alanine) couldn’t be fully built, leaving many gaps near the RuvC active site. In emd-0584 map, the HNH domain exhibits the lowest local resolution in the entire atomic model, built as a poly(Ala) model in the PDB reported for 6o0y (Zhu et al., 2019). In fact, there is no sidechain information anywhere in the HNH domain of that structure. It also appears that the HNH domain makes extensive new interactions with the displaced recognition (Rec) II domain whose local resolution was so low that this domain remained unbuilt. With the Rec II domain unbuilt or only partially reconstructed, one could conclude mistakenly that the HNH active site and the RNA/DNA duplex bound to Cas9 are partially exposed to solvent, which is clearly not the case after the Rec II domain is appropriately placed. Likewise, without sidechains or/and with unfilled gaps, there is seemingly substantial solvent-accessible space available in the RuvC active site, which again would be an erroneous conclusion. Even with such an incomplete atomistic model, the surface representation of the RuvC-catalytically relevant complex reported for 6o0y clearly shows that the RuvC active site and the ntDNA strand are fully buried inside the enzyme (Figures 1, 2). Likewise, the entire RNA/DNA heteroduplex is largely encircled by the enzyme (Figures 1, 2). Experimentally, the resolution of these structures needs to be improved before we can confidently visualize the catalytic active sites to gain insights into a complete understanding of the molecular mechanisms of this enzyme. In the meantime, molecular dynamics (MD) simulations can provide more complete structural models with greatly improved resolution (Nierzwicki and Palermo, 2021; Wang et al., 2022a; Wang et al., 2022b).

Here, we formulate some mechanistic hypotheses that could be computationally addressed and directly compared with currently available experimental data. There are two kinds of dynamics relevant to functionality, including i) small local motions of the constituent domains responsible for rearrangements of catalytic residues required for activation and ii) large motions of domains necessary for the protein-nucleic acid complex assembly. Some of these dynamic properties have been addressed using MD simulations and NMR spectroscopy as well as single-molecule spectroscopy (Singh et al., 2016; Palermo et al., 2017a; Palermo et al., 2017b; Chen et al., 2017; Dagdas et al., 2017; Zuo and Liu, 2017; Singh et al., 2018; Newton et al., 2019; Palermo, 2019; East et al., 2020a; De Paula et al., 2020; Ray and Di Felice, 2020; Wang Y. et al., 2021; Nerli et al., 2021; Nierzwicki et al., 2021; Cofsky et al., 2022; Wang et al., 2022c). We focus on analysis of four structural models, including the apo structure (4cmp, determined at 2.62 Å resolution), the RuvC-catalytically relevant complex (5f9r), the HNH-relevant complex (6o0y), and a high-resolution catalytically inactive RNA/DNA complex (5b2r) at 2.0 Å resolution, which is the most complete atomic model (Jinek et al., 2014; Hirano et al., 2016; Jiang et al., 2016; Zhu et al., 2019). Collectively, studies recently reported have shown that Cas9 is intrinsically flexible with rotations of many relatively small domains as observed for independent molecules of the same complex in the crystal lattice. Those rotations are functionally important, particularly in the activation of two individual nucleases.

### Domain structures of Cas9 and domain rotations

Cas9 from *Streptococcus pyrogenes* has 1,368 amino acid residues comprising the two catalytic domains of RuvC and HNH, three recognition (Rec) domains of Rec I, Rec II, and Rec III, a PAM-interacting domain, and a bridge helix (Figure 1A) (Jinek et al., 2014; Hirano et al., 2016; Jiang et al., 2016; Zhu et al., 2019). Rec II is inserted inside the Rec I domain so it can move relative to the Rec I domain, acting as a separate domain. The HNH domain (residues 775–909) is inserted into the RuvC domain, near the RuvC active site, through two linker loops (L1 and L2) that connect the two nuclease domains, which control the two enzymatic activities (Figure 1A).

**FIGURE 3:**
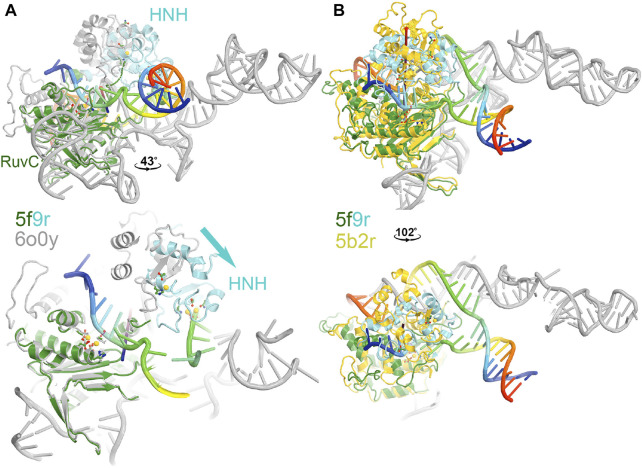
Two views of HNH domain rotations. **(A)** Between the RuvC and HNH catalytically relevant complexes of 5f9r and 6o0y. **(B)** Between the RuvC catalytically relevant and its inactive complexes of 5f9r and 5b2r. See supporting information for animation videos of the domain rotations (Supplementary Video S1).

In the RuvC-catalytically relevant complex, the nucleic acids adopt an R-structure with the HNH domain inserted between the RNA-DNA heteroduplex of the tDNA strand and the displaced ntDNA strand, which is completely buried inside the RuvC-HNH cleft (Figure 2). The HNH catalytic site is located 31 Å away from the scissile phosphate of the tDNA strand in the RuvC active complex. The interface at the RuvC-HNH cleft differs by a rotation of 102° relative to the two domains when comparing structures with and without the ntDNA bound (Figure 3, see Supplementary Video S1). After this rotation, the cleft between the RuvC and HNH domains remains closed. Following the HNH to RuvC rotational axis, the motion can be described as a twisting motion, like the motion of recombination reactions for exchanging two DNA strands, which cut two DNA strands, rotate them, and rejoin them. Well known examples of this kind of twisting motion include the resolution of Holliday junctions by RuvC and recombination of γδ resolvase (Ariyoshi et al., 1994; Li et al., 2005; Gorecka et al., 2013).

The HNH domain rotates differently relative to the RuvC domain when comparing the RuvC and HNH catalytically relevant complexes, both of which contain the ntDNA strand. However, one has an uncleaved ntDNA substrate ready for its cleavage while the other has a cleaved ntDNA product in the post-cleavage state. The transition between those two states involves an opening and closing motion of the RuvC-HNH domain cleft, with a relative rotation of the HNH domain by 43°. This motion is accompanied by a rotation of the Rec II domain, as the emd-0548 map (corresponding to the 6o0y coordinates) clearly shows that an extensive interface is formed between the HNH and Rec II domains so that the HNH catalytic active site is also buried in the HNH catalytically relevant complex.

MD simulations including enhanced sampling have been applied to characterize large conformational changes in the HNH domain of Cas9 (Palermo et al., 2017a). It has been shown that conversion to the pre-catalytic HNH state (H840 locates ∼15 Å from target scissile phosphate) (5f9R) from the inactive state (∼30 Å) (4un3) involves an ∼ 180° rotation around itself while employing critical H-bond interactions between the L2 loop (906–918) and the guide RNA:tDNA or gRNA:tDNA hybrid (Anders et al., 2014; Jiang et al., 2016; Palermo et al., 2017a). Such a large conformational change may not happen in a single step after the completion of tDNA:ntDNA unwinding. It is likely a stepwise progression during double strand separation. After formation of the pre-catalytic state, HNH can easily adopt the catalytically competent state (H840 docked at ∼ 4–6 Å from target site) by employing a high degree of dynamics of HNH and L2–ntDNA interactions. Docking of HNH at the target site is also associated with a large scale opening of the Rec II—Rec III (residues 497–713) clefts. Specifically, highly correlated dynamics of HNH and Rec III are observed in MD simulations, suggesting a central role of the Rec domain in gRNA:tDNA “sensing” and subsequently modulating the HNH positioning relative to the target site (Palermo et al., 2018). The Rec domains also undergo large scale conformational changes with respect to the nuclease domains while moving from apo (4cmp) to the gRNA bound states (4zt0) (Jinek et al., 2014; Jiang et al., 2015). In fact, MD simulations reported that solvent exposure of the arginine-rich bridge helix is crucial for RNA recruitment and further accommodation by formation of a positively charged RNA-binding cavity (Palermo et al., 2017a).

### RuvC catalytic site

**FIGURE 4:**
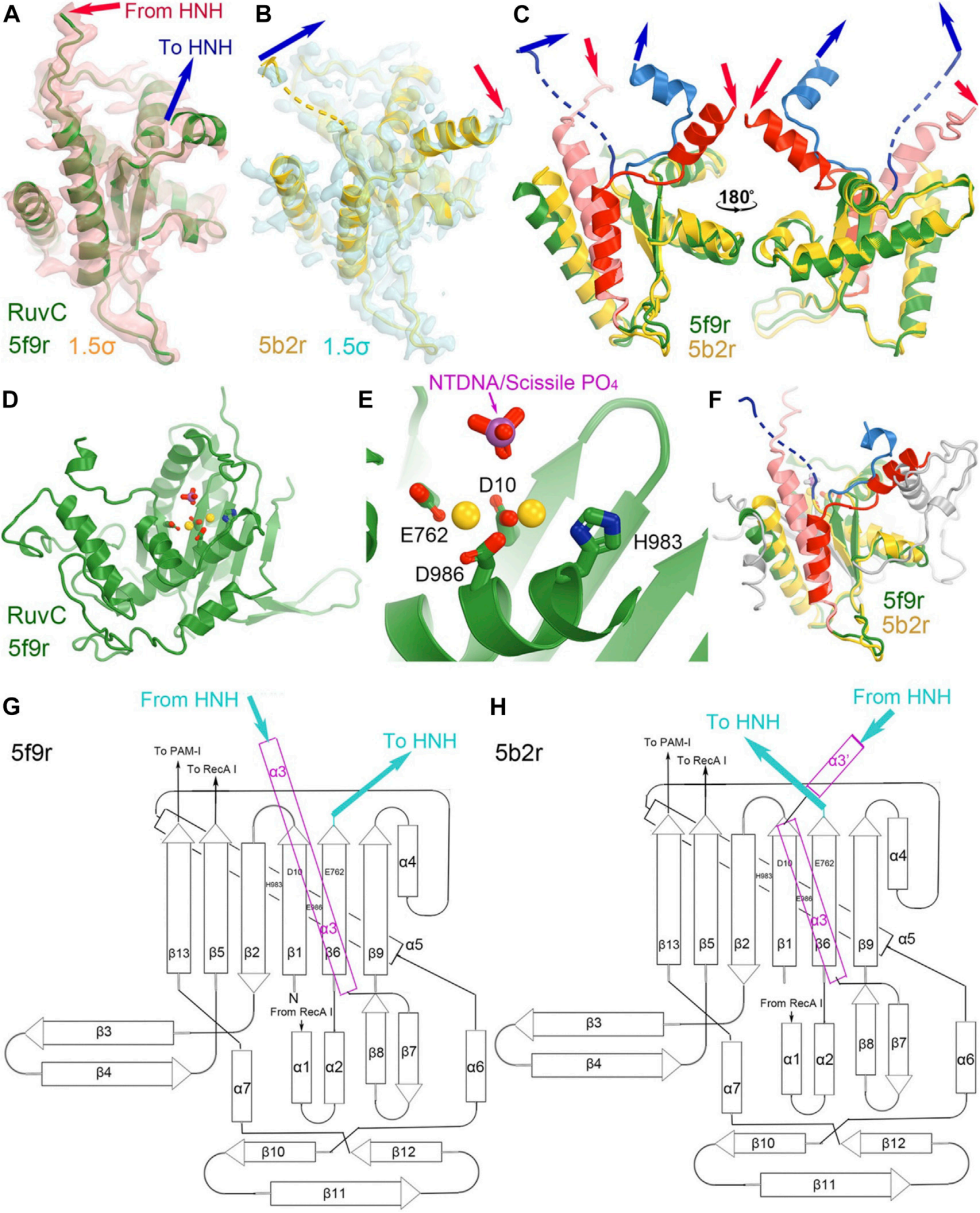
Structures of the RuvC domain in the RuvC catalytically relevant (5f9r) and catalytically inactive (5b2r) complexes. **(A)** Crystallographic electron density maps for a portion of the 5f9r RuvC structure contoured at 1.5σ. **(B)** Crystallographic electron density maps for a portion of the 5b2r RuvC structure. **(C)** Two views of the superposition of part of the two structures. **(D,E)** A complete view of the entire RuvC domain and zoom-in view of the RuvC catalytic site, with two metal ions computationally modeled. The ntDNA scissile phosphate is shown in magenta and red. **(F)** Superimposition of the complete RuvC domains of the two structures with the catalytic residues indicated. **(G,H)** local topological drawings of the RuvC domain in the two structures.

The RuvC catalytic residues, including D10, E762, E986, and H983, were initially inferred from closely related structures and subsequently confirmed by site-directed mutagenesis (Figure 4) (Tang et al., 2021). Those residues are thought to bind two Mg^2+^ ions with binding affinity of 1.6 mM and 5.9 mM, respectively, although these specific binding sites have yet to be structurally characterized. Structural biologists often use Mn^2+^ in crystallographic electron density maps or cryo-EM derived electrostatic potential maps for metal ion identification (Leonarski et al., 2017; Wang J. et al., 2021). Nonetheless, based on the proximity between catalytically essential residues and the putative scissile phosphate of the ntDNA strand, it can be ascertained that the 5f9r structure represents a RuvC-catalytically relevant complex. The two metal ions can be approximately placed between these residues and between the enzyme and the scissile phosphate of the ntDNA strand, even though the resolution of the structure is not sufficiently high as necessary for their direct identification. Given the approximate 31 Å distance of the catalytic residues of the HNH domain to the putative scissile phosphate of the tDNA strand in this complex, HNH is clearly not positioned for the ntDNA cleavage.

The connecting linker loops between the RuvC and HNH domains have been well defined in electron density maps of the 5f9r complex. L1 comprises a short three-residue, extended structure (E766-Q768) plus a short α-helix (T769-R778) before entering the HNH domain while L2 includes an extended loop (S909-I917) and a long α-helix (I917-N940) connecting the RuvC domain. These two linker loops change local structures and swap positions in the HNH-relevant complex between the 5f9r and 6o0y complexes, related by a twisting domain motion (Figure 4). L1 becomes an extended strand structure (after β 6) and L2 breaks into two helices (α3'+α3) in the 6o0y complex. From the local topology of the RuvC domain we observe that the two linker regions are placed after the β6 strand and within α3 helix (Figures 4G, H). Therefore, rotation of the HNH domain relative to the RuvC domain involves extensive local remodeling of linker regions and their interacting partners (Bravo et al., 2022).

### HNH catalytic site

**FIGURE 5:**
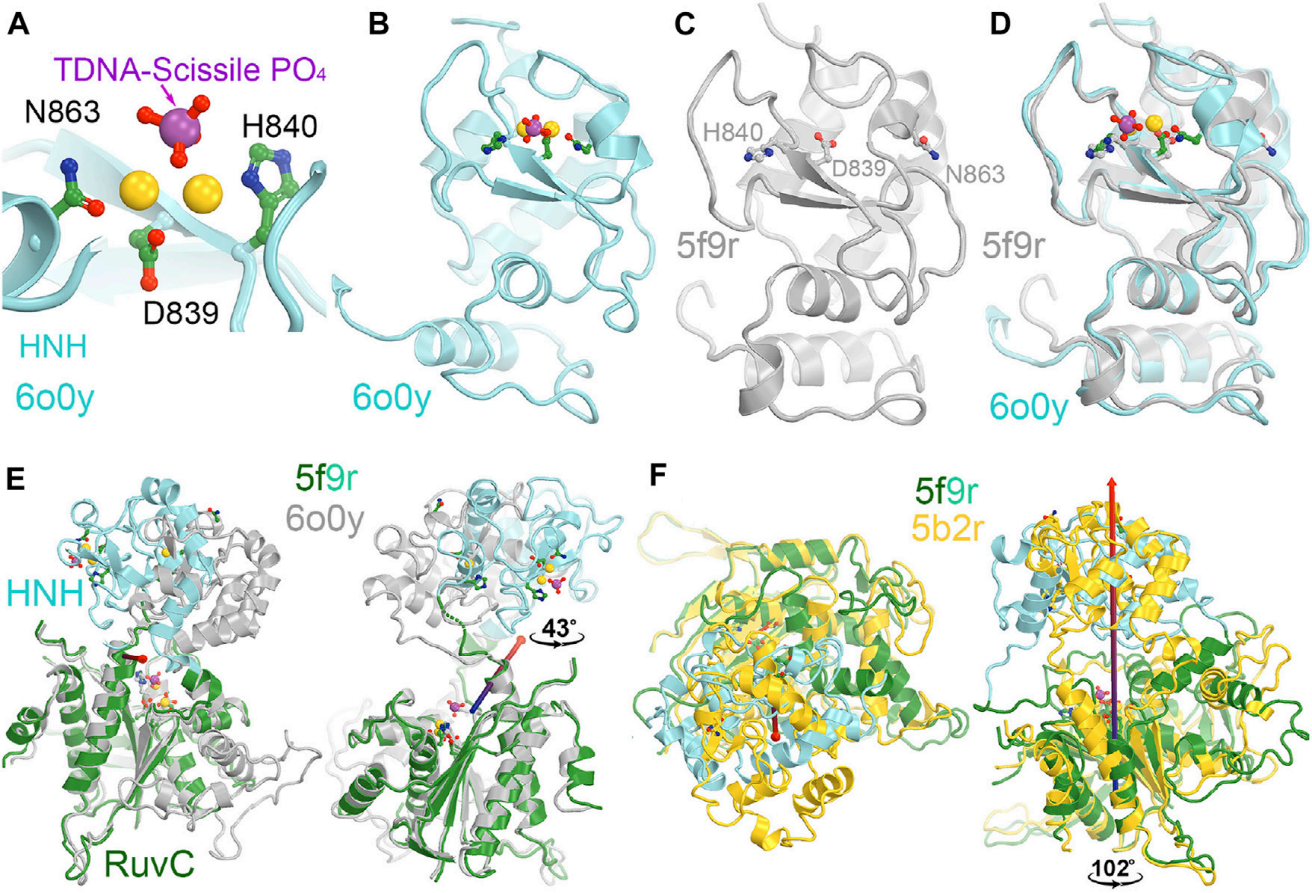
HNH catalytic site. **(A)** A close-up view of the HNH active site in the catalytically relevant complex of 6o0y with one Mg^2+^ ion computationally modeled. **(B)** A zoomed-out overall view of the entire HNH domain of 6o0y. **(C)** An overall view of the inactive 5f9r HNH domain. Note that N863 points away from H840. **(D)** Superposition of the 6o0y and 5f9r HNH domains. **(E)** Two views of superpositions of the RuvC domain to see relative rotations of the HNH domain between the 5f9r and 6o0y complexes. Rotation axis is indicated by the arrow. **(F)** Two views of superpositions of the RuvC between the 5f9r and 5b2r complexes.

The catalytically essential residues for the HNH domain have been identified to be D839, H840, and N863 (Figure 5) (Gasiunas et al., 2012; Zuo et al., 2019; Tang et al., 2021). The catalytic mechanism for the tDNA cleavage by HNH was proposed to involve a single metal ion, rather than two metal ions (Raper et al., 2018). The binding affinity of this metal ion is also relatively weaker than 6 mM (*K*_d_) (Raper et al., 2018). However, there is no biochemical evidence directly supporting binding of the second divalent metal during the catalytic process. Assuming that a single ion is involved, it would likely bind between the reoriented N863 sidechain and D839. Before the HNH domain becomes catalytically active, the N863-containing loop residues adopt a very different conformation from the active form. In the inactive conformation, the sidechain of N863 points away from the metal ion binding site, and the N863 backbone is displaced 3.4 Å away from H840 i.e., 9.0 Å in the HNH active conformation to 12.4 Å in the HNH inactive conformation. Aside from the conformation of the N863-containing loop, the overall backbone structure is similar in the two conformations.

Recent MD simulations of an isolated HNH domain in the wild-type enzyme and three single Lys-to-Ala mutated enzymes show that the HNH domains remain in an inactive conformation in the absence of DNA substrate in an isolated HNH domain as determined by the N863 location and its backbone conformation (Wang et al., 2022c). There is no direct evidence of spontaneous conversion to the active conformation in the absence of substrate outside the intact Cas9 enzyme in MD simulations. That study also showed that because of a possible higher frequency of spontaneous conversion from inactive conformations to a state that is very close to the activated state, the wild-type enzyme is more dynamic than the three single mutants, which is consistent with the observation that the wild-type enzyme is more promiscuous for substrate selection than the three alanine mutants (Wang et al., 2022c). This was further supported by NMR studies of the isolated HNH domain, where the Y836-containing loop (which mediates interactions with the Rec II domain and the tDNA strand, both playing essential roles during activation of the HNH domain) exhibits increased flexibility in the wild-type enzyme relative to three alanine mutations (Wang et al., 2022c). It is noted that Y836 is only two residues away from the two catalytic residues of D839 and H840, and its interacting partner D861 of the Y836-D831 pair in a second conformation identified is only one residue away from the third catalytic residue H863 (Wang et al., 2022c). Moreover, the Y836-containing loop mediates interactions with Rec II domain and interacts with the tDNA strand that is only about two nucleotides 3′ away from the scissile phosphate. All these observations highlight the importance of Y836 in regulation of the HNH nuclease activity. Analysis of large-scale relative rotations between the HNH and RuvC domains (Supplementary Video S2) infers new potential functions of these surface Lys residues through regulation of the domain motions and stability of the HNH domain. We note that K548 is part of the RuvC active site. By using a single divalent metal ion for catalysis, instead of the classic two divalent metal ions, alignment of function groups (particularly, the general base and general acid for catalysis) would be more important and can be highly regulated.

### Dual activation roles of the HNH domain

**FIGURE 6:**
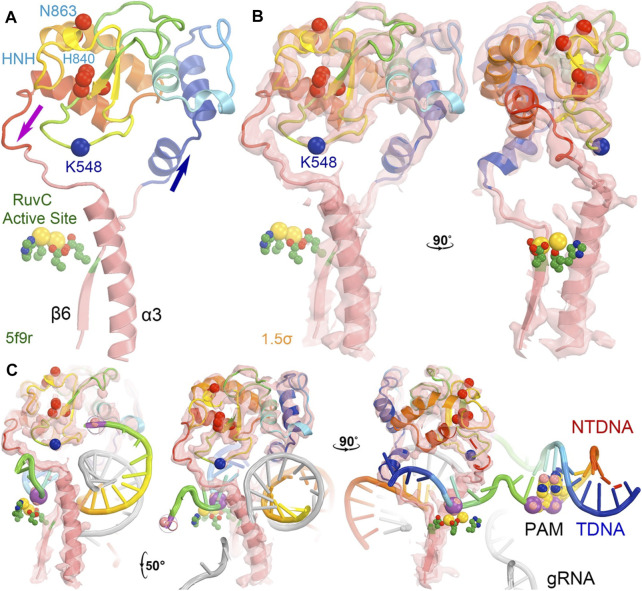
The HNH domain serves as a part of the RuvC active site in the 5f9r structure. **(A)** The HNH domain is inserted between the β6 stand and α3 helix near the RuvC active site (green side chains plus two modeled Mg ions in gold). The location of K548 (which was built as an alanine residue as well as many other residues also as alanine residues including K510 and some HNH catalytic residues). **(B)** Two orthogonal views of σ_A_-weighted F_o_–F_c_ ED maps retrieved from the PDB contoured at + 1.5σ. **(C)** Three views of the maps with modeled nucleic acids (which had much stronger ED values).

The entire backbone of the HNH-RuvC region was reasonably well defined in the crystallographic electron density maps, although the loop containing K848 was built as a poly(alanine) model and so were many other regions of the HNH domain in the 5f9r structure (Figure 6) (Jiang et al., 2016). This loop makes extensive interactions with L2 before L2 connects to α3 helix of RuvC. The K848-containing linker loop also wraps around the ntDNA strand at the scissile phosphate (Figure 6C). The K848 Cα coordinate is approximately located at equal distance of 9.4 Å to both the tDNA and ntDNA strands (at phosphate group and nucleobase). Therefore, the K848-containing loop is part of the RuvC active site. After cleavage of the ntDNA strand, the K848-containing loop no longer maintains strong interactions with L2 and the cleaved ntDNA strand, so the entire HNH domain rotates away from the RuvC active site by 43° to be closer to the tDNA strand as observed in the 6o0y complex structure (Zhu et al., 2019).

**FIGURE 7:**
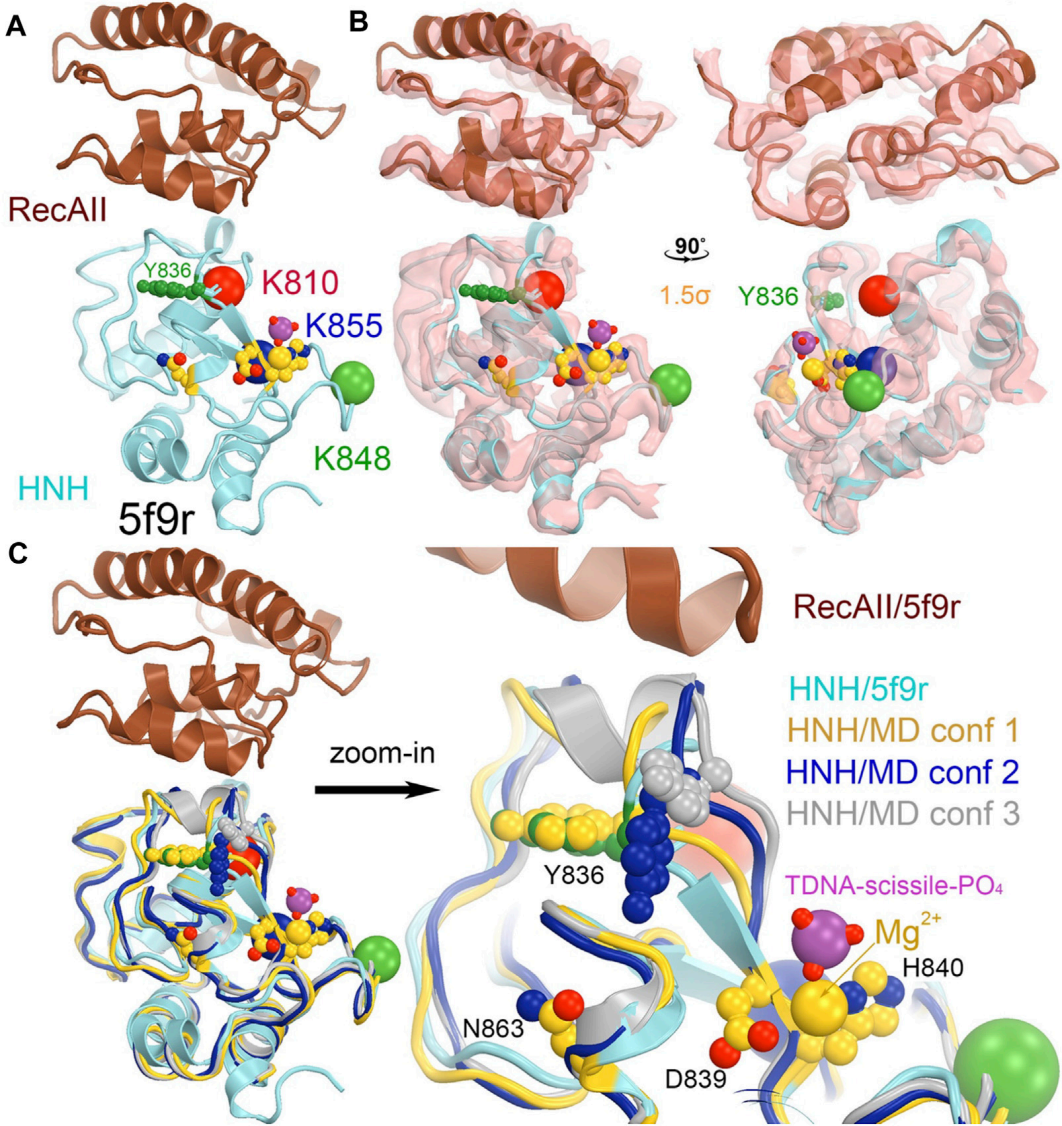
Locations of three MD-derived HNH conformations at the HNH-Rec II interface. **(A)** HNH (cyan)-Rec (brown) interface from the 5b9r complex. **(B)** Crystallographic electron density map (contoured at 1.5σ) for the 5f9r complex in two orthogonal views. **(C)** Alignment of the MD-derived three HNH conformations (gold, blue, and silver) with the 5f9r HNH (cyan) structure. The tDNA scissile phosphate is shown in magenta, Mg^2+^ ion in gold, as well as three catalytic residues (D839/H840 and N863) in an inactive conformation.

**FIGURE 8:**
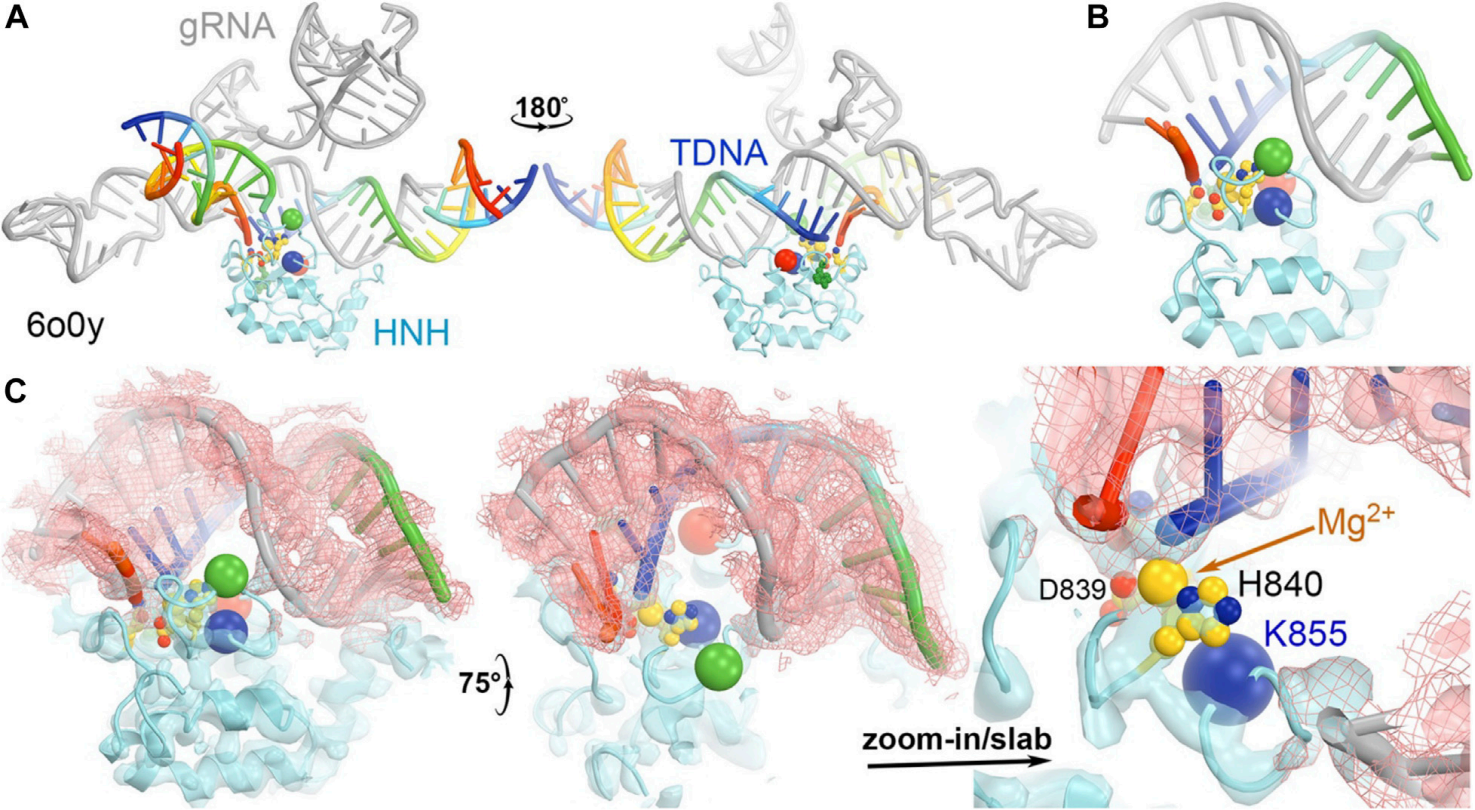
**(A)** Two front and back views of the HNH domain from the HNH-activated Cas9 complex of 6o0y. **(B)** Close-up view of the HNH binding at the minor groove of the RNA/DNA duplex. **(C)** Three views of the complex superposed with emd-0584 contoured at 6σ (cyan isosurface and salmon isomesh) and 12σ (salmon isosurface).

In the 5f9r complex, the Y836-containing loop makes extensive interactions with the Rec II domain, as expected in the 6o0y structure upon examination of its emd-0854 map, even though the Rec II domain wasn’t built in the atomic model and thereby not included in the coordinate file (Figure 7) (Jiang et al., 2016; Zhu et al., 2019). The tDNA strand in the 6o0y structure binds to the interface of the HNH and Rec II domains (Zhu et al., 2019). Three distinct conformations of the Y836-containing loop observed in the MD simulations of an isolated wild-type HNH domain are located near the HNH-Rec II domain interface in the 5f9r complex (likely in the 6o9y complex) and near the tDNA strand that directly connects to the HNH catalytic site *via* the tDNA-induced activation of the HNH activation (Figure 8). Given the fact that three Lys residues (K810, K848, and K855) are inserted into the minor groove of the RNA/DNA heteroduplex, they provide stabilization for the complex formation. By mutating them to Ala, the mutant HNH enzymes become destabilized for the unregulated complex formation and prevent the DNA substrate-independent activation of the wild-type enzyme.

### Catalytic mechanisms of DNA cleavage

The RuvC and HNH domains of Cas9 cleave the ntDNA strand (utilizing two Mg^2+^ ions) and tDNA strand (involving a single Mg^2+^ ion), respectively. Crystallographic studies reported the most relevant catalytic state of Cas9 with Mn^2+^ as an alternative to Mg^2+^ in the RuvC domain (Palermo et al., 2017a). The Mn^2+^ ions were well coordinated in the catalytic core by highly conserved residues, including D10, D986, E762 and H983. Ab *initio* MD simulations that replaced Mn^2+^ by Mg^2+^ have shown that a conformational rearrangement of H983 could make that residue function as a general base (Palermo, 2019). Recent studies led to a proposal of a catalytic mechanism based on high-level quantum mechanical methods and first-principles MD simulation (Brunk et al., 2011). A mixed quantum mechanics/molecular mechanics (QM/MM) approach was combined with free energy-calculation methods, revealing that a water molecule bridges between H981 and the scissile phosphate. In that mechanism, H981 acts as the activator and accepts a proton from the nucleophilic water followed by the simultaneous breakage of O3′-P_scissle_ bond and formation of P_scissle_-OH_water_ bond according to an associative S_N_2 mechanism (Casalino et al., 2020). The role of H983 as a nucleophilic activator is in accordance with mutation data revealing a hampered cleavage of the ntDNA strand when H983 is mutated to alanine. Further in-depth discussion can be found elsewhere (Nierzwicki et al., 2021; Patel and Palermo, 2022; SahaAshan et al., 2022).

The tdDNA cleavage by RuvC involves an intricate conformational rearrangement of the HNH domain but hasn’t yet been fully resolved mechanistically due to the lack of sufficiently high-resolution structural data. The studies of the catalytic mechanism in HNH have been based on the comparison to its closest analogue, the T4 endonuclease VII. The analysis suggested the role of D861, D839 and N863 in coordinating Mg^2+^ for catalysis, whose coordination sphere is saturated by nucleophilic water (Sternberg et al., 2015; Palermo et al., 2017a; Zuo and Liu, 2017). This suggestion was supported by several X-ray structures that captured different catalytic states of HNH with D861 pointing towards D839 (Anders et al., 2014; Jinek et al., 2014; Nishimasu et al., 2014; Huai et al., 2017). With D861, the active site could potentially bind two metal ions instead of one, for which functional role remains unclear. Recent reports of catalytically active HNH structures exhibited a different configuration of the active site where N863 (rather than D861) coordinates Mg^2+^ and forms a catalytic triad with D839 and H840 (Zhu et al., 2019; Bravo et al., 2022). An alternative catalytic mechanism was also proposed where N863 does not engage in the metal coordination and the catalytic water comes from the second shell of metal ion coordination (Zhao et al., 2020). *Ab initio* QM/MM studies of the tDNA cleavage revealed the activation of nucleophilic water by H840, followed by catalysis through a concerted associative mechanism similar to RuvC (Nierzwicki et al., 2022). Interestingly, the water molecule coordinating the Mg^2+^ ion was seen to shuttle a proton from K866 to the DNA O3’ to form the final product, suggesting a possible catalytic role of K866. Despite having little effect on the p*K*_a_ of the catalytic H840 when measured by NMR, mutation of K866 to alanine (K866A) showed a remarkable reduction in enzymatic activity (Nierzwicki et al., 2022). Thus, the quantum mechanics level studies, combined with NMR and biochemical studies, have assisted in the identification of the critical second-shell residues in metal-dependent enzymes.

### Domain motions in Cas9 in comparison with DNA polymerases

The CRISPR systems of bacteria must be able to discriminate between the target foreign DNA duplexes from closely related DNA duplexes as well as its own genome encoded DNA duplex. How they achieve a high degree of discrimination remains only partially understood with multiple possibilities identified. One such mechanism is the CRISPR GUARD mechanism, which stands for Guide RNA Assisted Reduction of Damage and uses a special gRNA to protect off-target DNA duplexes (Coelho et al., 2020). A critical feature in discrimination mechanism is to regulate the activation steps of both RuvC and HNH activities, which remain inactive for off-target DNA duplexes but become active only for on-target DNA duplexes.

This on-off regulation of Cas9 for on-target cognate and off-target non-cognate substrates is reminiscent to a high degree of base selectivity exhibited by replicative DNA polymerases, which catalyze efficient nucleotide incorporation only for the cognate substrates of Watson-Crick base paired dNTP but reject noncognate non-Watson-Crick base paired dNTPs (Kunkel and Bebenek, 2000; Xia and Konigsberg, 2014). In both Cas9 and DNA polymerases, large conformational changes are involved in the activation step only for cognate substrates (but not for non-cognate substrates), all of which are commonly known as induced-fit. This is also described as general allostery in regulation, which extends beyond the original term describing the changing affinity of oxygen binding through subunit communications in human tetrameric hemoglobin. Experimentally, it is relatively easy to visualize the relative stable enzyme-substrate or enzyme-product complex during the action of catalysis but is nearly impossible to do so for any enzyme/non-cognate substrate complex during the action of being rejected. In this sense, computational biophysics in conjunction with biochemical and biophysical experiments could provide valuable mechanistic insights into substrate specificity and allostery at the detailed molecular level as discussed here (Wodak et al., 2019; East et al., 2020b; Nierzwicki et al., 2021; Belato et al., 2022; Nierzwicki et al., 2022).

Without large conformational changes, the enzyme simply lowers the free energy barrier of the transition state of the catalyzed elemental reaction without altering its equilibrium (Kraut, 1988), and thus can’t provide a high degree of discrimination between cognate and non-cognate substrates due to only small geometrical and chemical differences between them. The forward and reverse rates of large conformational changes that are connected to the transition state are modulated very differently by cognate and non-cognate substrates. It is the large conformational changes (aka, conformational checkpoints) that can block non-cognate substrates from going forward to the transition state while directing them onto alternative pathways that are eventually led to their release. In the reaction catalyzed by DNA polymerases, the product pyrophosphate is continuously removed from the active site and hydrolyzed so that the reverse pyrophosphorylysis reaction never plays a significant role. After mis-insertion of non-Watson-Crick base paired nucleotides, some polymerases retain the product pyrophosphate in the active site longer for the likelihood of pyrophosphorylysis (Xia et al., 2013; Xia and Konigsberg, 2014; Wang and Konigsberg, 2022). In this aspect, Cas9 also has some unique properties because Cas9 is a single turnover enzyme that differs from processive DNA polymerases and kinetic studies have shown that R-loop formation (i.e., the association of DNA to the RNA-bound Cas9 complex) is rate-limiting for DNA cleavage (Gong et al., 2018; Raper et al., 2018).

The interactions between Cas9 and the RNA/DNA heteroduplex and between Cas9 and gRNA duplex are mainly electrostatic. The binding affinity of the dsDNA duplex with the DNA-RNA base pairing is about 3 *n*M (Raper et al., 2018). The rate limiting step is the formation of a R-loop structure in which the HNH domain is inserted between the displaced ntDNA and the newly formed tDNA-RNA heteroduplex (Gong et al., 2018; Raper et al., 2018). This process involves a swiveling motion of the HNH domain relative to the RuvC domain by 102° as characterized in this review. This motion moves the ntDNA strand next to the tDNA/RNA heteroduplex outside the HNH domain to the opposite side of the HNH domain to bury the tDNA strand inside the HNH active site and thus alters the topology of the R-loop structure similarly to many other topology-modifying enzymes such as topoisomerases, γδ resolvase, and RuvC (Ariyoshi et al., 1994; Li et al., 2005; Gorecka et al., 2013). This ensures that Cas9 wouldn’t pick up any other preformed, unrelated R-loop structure such as RNA polymerase transcription intermediates.

The Rec domains of Cas9 form large nucleic acid-binding sites (Eggleston and Kowalczykowski, 1991; Kowalczykowski, 1991). A major difference between Cas9 and RecA is the mechanism of strand displacement during the formation of R-loop structure, which is exclusively driven by extensive interactions in Cas9 between gRNA and the enzyme, particularly after the PAM-interacting domain of Cas9 recognizes the PAM sequence. It doesn’t require the free energy from ATP binding or hydrolysis as in RecA (Eggleston and Kowalczykowski, 1991; Kowalczykowski, 1991). The higher thermodynamic stability of cognate RNA:DNA hetero-duplex substrates, compared to a canonical B-DNA, likely favors R-loop formation. Stepwise interrogating DNA sequence ensures the fidelity of the RuvC cleavage during R-loop formation (Cofsky et al., 2022).

### Domain rotation mechanisms as the means for allosteric regulation

The videos provided in supporting information can assist our understanding of different functional states since they describe structural differences among different complexes. The videos haven’t included remodeling of linker regions between the RuvC and HNH domains (i.e., those regions were deleted for visualization of conformational differences).

All-atom MD simulations, in combination with network models derived from graph theory, have shown that the binding of PAM induces a population shift and highly coupled motions of HNH and RuvC, showing a typical allosteric response that is in line with previous biochemical studies (Palermo et al., 2017b). PAM binding has an important role in the formation of an optimal allosteric network, when compared to the system without PAM. These data suggest that PAM acts as an “allosteric effector” in Cas9 systems. The analysis of the allosteric pathways revealed that the communication between the HNH and RuvC domains flows through the L1/L2 loops, reported as “signal transducers” (Jiang et al., 2016). Experimental modifications in the L1/L2 loops led to the development of a Cas9 variant with improved specificity (LZ3-Cas9) (Schmid-Burgk et al., 2020). Furthermore, other mutations in the central nodes of the communication, also reported increased specificity, i.e., the K775A and R905A mutations in the eCas9 and HypaCas9 variants, respectively (Slaymaker et al., 2016; Chen et al., 2017).

NMR relaxation dispersion experiments and MD simulations have shown that the core residues of HNH form an allosteric pathway connecting the Rec domains to the HNH and RuvC catalytic sites, where slow (millisecond) dynamics are critical for the signal transmission that mediates the communication of DNA binding information from the Rec domain to the nuclease sites (East et al., 2020a). Surprisingly, in a thermophilic variant (GeoCas9), this interdomain signaling was replaced by faster (nanosecond) dynamics when compared to SpCas9 (Belato et al., 2022). The three individual mutations of K810A, K848A, and K855A were also investigated in the HNH allosterism (Slaymaker et al., 2016). These mutations were revealed to interrupt the main allosteric pathway connecting Rec to RuvC (Nierzwicki et al., 2021). Interestingly, for the three single mutants, the mutation that strongly perturbed the signal transfer also achieved the highest specificity, indicating a direct link between changes in the allosteric network and the increase in the Cas9 specificity (Nierzwicki et al., 2021). These all-atom MD simulations show that the allosteric regulation in CRISPR-Cas9 can be used to improve the system specificity. As noted above, early computational studies, using biasing methods and accelerated MD simulations (Palermo et al., 2017a; Palermo et al., 2018), traced the swiveling motion of 102° of the HNH domain relative to the RuvC domain within the Cas9 complex, and reported the energetic barriers during this transition as follows. Analysis of the intermediate steps involved calculation of the individual atomic B-factors from MD-derived electron density (ED) maps (or electrostatic potential maps, MD-ESP maps). Atomic B-factors are linearly related to squares of fluctuations (*B* = 8π^2^|Δr|^2^, where |Δr| is root-mean-squares fluctuation). As a result of this analysis, the structure with the lowest free energy has the smallest mean atomic B-factors with the deepest free energy well. Unstable structures may never reach an equilibrium state and the corresponding MD-derived ED maps are often uninterpretable. The same computational approaches could be applicable to other domain-rotations problems such as DNA duplex strand exchange reactions within dimer of dimers in γδ resolvase and RuvC in resolution of Holliday junction (Ariyoshi et al., 1994; Li et al., 2005; Gorecka et al., 2013). Domain rotation and allosteric regulation are also critical for the regulation of off-target substrates. Indeed, the rotation of the HNH domain toward activation can be modulated allosterically (Dagdas et al., 2017). Enhanced simulation methods have shown that the rotational activation of HNH is tightly dependent on the presence of DNA base pair mismatches within the RNA:DNA hybrid. Depending on their position and nature, DNA mismatches can induce an opening of the RNA:DNA hybrid, and lock the catalytic HNH domain in an inactive state (Ricci et al., 2019; Mitchell et al., 2020). These findings were corroborated by X-ray structures of Cas9 bound to off-target substrates, providing a structural rationale for the off-target activity of Cas9 and contributing to the design of guide RNAs and off-target prediction algorithms (Pacesa et al., 2021).

**FIGURE 9:**
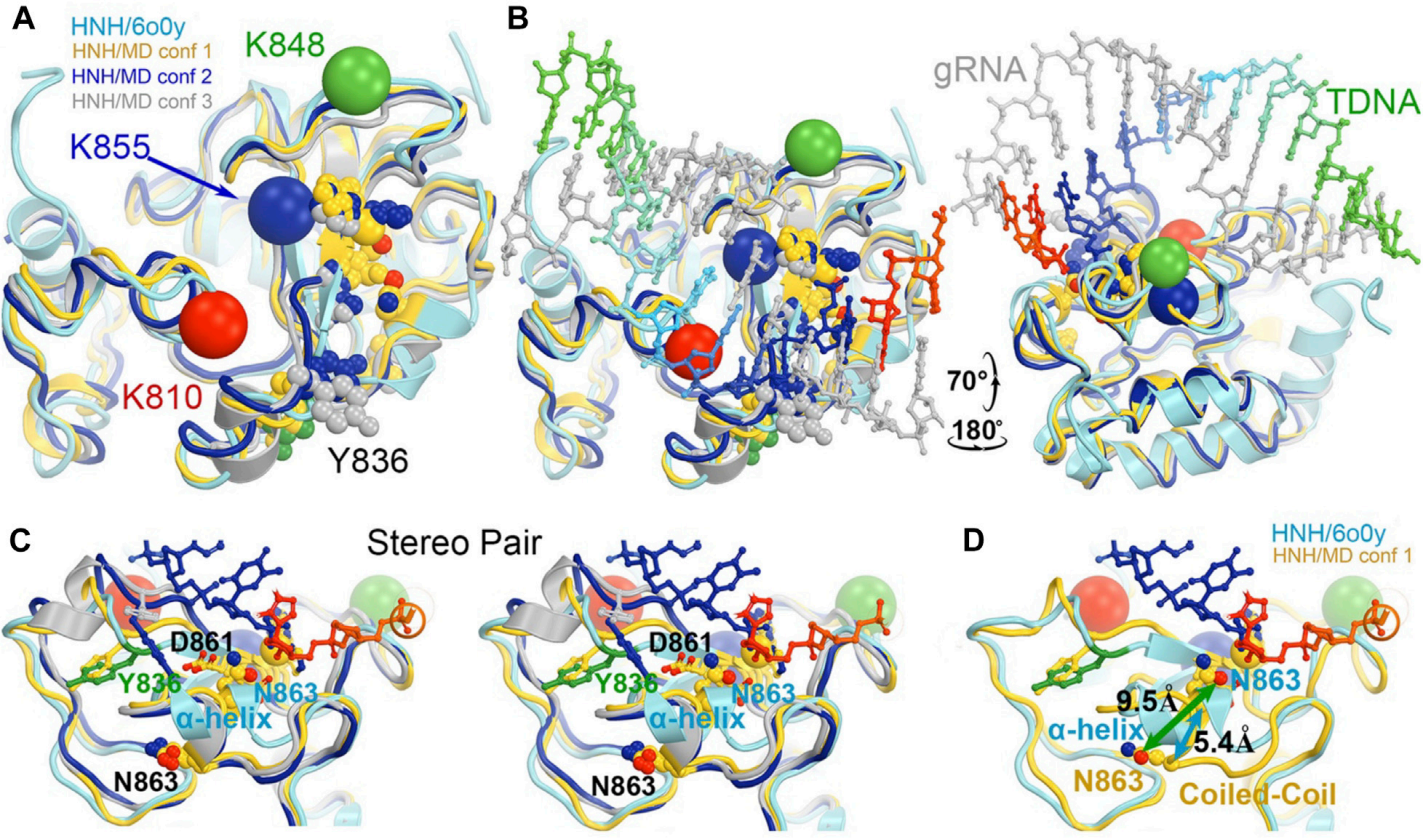
Potential roles of three MD-derived HNH conformations during activation of the HNH active site. **(A)** Alignment of the three MD-derived conformations of the HNH domain (gold, blue, and silver for conformations 1, 2, and 3, respectively) superimposed onto the 6o0y experimental structure (cyan). Locations of three Lys-to-Ala mutations are shown in large spheres at Cα: K810, red, K848, green, and K855, blue. Metal ion is in medium-size gold sphere. Y836, D861, N863, and H840 residues are shown. **(B)** Two views of the superposition in the presence of the RNA/DNA duplex. **(C)** Stereodiagram of a close-up view for showing the relationship of the Y836/D861/N863 three residues **(B)**. **(D)** A conversion of coiled-coil to α-helix results in a large displacement of N863 (5.4 Å at Cα and 9.5 Å at Oε1). See Supplementary Video S2 for locations of three mutants relative to domain rotations.

Divide-and-conquer methods, as have been demonstrated experimentally (East et al., 2020b; De Paula et al., 2020; Nerli et al., 2021; Nierzwicki et al., 2021; Nierzwicki et al., 2022), could be applied to break the extended system into small working parts to study the conversion of the HNH from the inactive to the active conformation. Given the known locations of K810, K848, and K855 at the minor groove of the RNA/DNA heteroduplex in the activated HNH complex, MD simulations could provide atomic resolution structures showing how these sidechains recognize the heteroduplex for HNH activation. We also envision using the minimized version of the HNH complex as outlined in Figure 9 to study the individual steps of the activation of HNH and cleavage processes.

## Concluding remarks

Cas9 is a highly dynamical biomolecule and exists in many different functional states in order to perform biological functions optimally and efficiently. To fully understand the specific function of each state, one must apply integrative approaches combining computational and experimental methods. Under a single experimental condition, three different functional states of Cas9 have already been detected to coexist simultaneously (PDB IDs of 6o0x, 6o0y, and 6o0z) (Zhu et al., 2019), as is similarly observed for ribosomes that also exhibit multiple functional states (both active and inactive) (Dashti et al., 2014; Jahagirdar et al., 2020; Poitevin et al., 2020). Overall, molecular simulations identified two distinct, nearly orthogonal motions of HNH relative to RuvC that are essential for substrate selection and cleavages. A back-and-forth twisting motion of the HNH domain about 102° moves non-target DNA strand from outside of Cas9 into the RuvC active site buried inside Cas9 during R-loop formation for cleavage of the non-target DNA strand and subsequent release of the cleaved ntDNA product. A swinging motion of the HNH domain about 43° aligns the HNH active site to the target DNA strand for cleavage of the target DNA strand and release of the cleaved tDNA product.

Experimentally determined structures of macromolecules often correspond to equilibrium states under well-defined experimental conditions, which can be simulated by equilibrium structures obtained by MD simulations (Wang et al., 2022a; Wang et al., 2022b; Wang et al., 2022c). Moreover, MD simulations can extend the resolution of existing cryo-EM maps when particles are divided into different functional state (Nierzwicki and Palermo, 2021). In fact, MD simulations can even provide details of the dynamics of interconversion between different functional states. The resulting movies can provide guidelines for designing experiments (and/or certain mutant enzymes) to enrich specific intermediates that could be more readily detected experimentally. The combination of computational and experimental approaches is therefore expected to be essential to design Cas9 with novel functionalities.

## Abbreviations

Cas9, CRISPR-associated protein 9; CRISPR, clustered regularly interspaced short palindromic repeat; ED, electron density; ESP, electrostatic potential; HNH, Histidine-asparagine-histidine motif endonuclease; MD, molecular dynamics; ntDNA, non-target DNA; PAM, protospacer adjacent motif; gRNA, guide RNA; Rec, recognition lobe or domain; tDNA, target DNA.

## References

[B1] Anders C. Niewoehner O. Duerst A. Jinek M. (2014). Structural basis of PAM-dependent target DNA recognition by the Cas9 endonuclease. Nature 513, 569–573. 10.1038/nature13579 25079318 PMC4176945

[B2] Ariyoshi M. Vassylyev D. G. Iwasaki H. Nakamura H. Shinagawa H. Morikawa K. (1994). Atomic structure of the RuvC resolvase: A holliday junction-specific endonuclease from *E. coli* . Cell 78, 1063–1072. 10.1016/0092-8674(94)90280-1 7923356

[B3] Belato H. B. D'Ordine A. M. Nierzwicki L. Arantes P. R. Jogl G. Palermo G. (2022). Structural and dynamic insights into the HNH nuclease of divergent Cas9 species. J. Struct. Biol. 214, 107814. 10.1016/j.jsb.2021.107814 34871741 PMC8917064

[B4] Bravo J. P. K. Liu M. S. Hibshman G. N. Dangerfield T. L. Jung K. McCool R. S. (2022). Structural basis for mismatch surveillance by CRISPR-Cas9. Nature 603, 343–347. 10.1038/s41586-022-04470-1 35236982 PMC8907077

[B5] Brunk E. Ashari N. Athri P. Campomanes P. de Carvalho F. F. Curchod B. F. (2011). Pushing the frontiers of first-principles based computer simulations of chemical and biological systems. Chim. (Aarau) 65, 667–671. 10.2533/chimia.2011.667 22026176

[B6] Casalino L. Nierzwicki L. Jinek M. Palermo G. (2020). Catalytic mechanism of non-target DNA cleavage in CRISPR-Cas9 revealed by *ab initio* molecular dynamics. ACS Catal. 10, 13596–13605. 10.1021/acscatal.0c03566 33520346 PMC7842700

[B7] Chen J. S. Dagdas Y. S. Kleinstiver B. P. Welch M. M. Sousa A. A. Harrington L. B. (2017). Enhanced proofreading governs CRISPR-Cas9 targeting accuracy. Nature 550, 407–410. 10.1038/nature24268 28931002 PMC5918688

[B8] Coelho M. A. De Braekeleer E. Firth M. Bista M. Lukasiak S. Cuomo M. E. (2020). CRISPR GUARD protects off-target sites from Cas9 nuclease activity using short guide RNAs. Nat. Commun. 11, 4132. 10.1038/s41467-020-17952-5 32807781 PMC7431537

[B9] Cofsky J. C. Soczwek K. M. Knott G. J. Nogoles E. Doudna J. A. (2022). CRISPR-Cas9 bends and twists DNA to read its sequence. Nat. Struct. Mol. Biol. 29, 395–402. 10.1038/s41594-022-00756-0 35422516 PMC9189902

[B10] Dagdas Y. S. Chen J. S. Sternberg S. H. Doudna J. A. Yildiz A. (2017). A conformational checkpoint between DNA binding and cleavage by CRISPR-Cas9. Sci. Adv. 3, eaao0027. 10.1126/sciadv.aao0027 28808686 PMC5547770

[B11] Dashti A. Schwander P. Langlois R. Fung R. Li W. Hosseinizadeh A. (2014). Trajectories of the ribosome as a Brownian nanomachine. Proc. Natl. Acad. Sci. U. S. A. 111, 17492–17497. 10.1073/pnas.1419276111 25422471 PMC4267381

[B12] De Paula V. S. Dubey A. Arthanari H. Sgourakis N. G. (2020). A slow-exchange conformational switch off-target cleavage by high-fidelity Cas9. BioRXiv. doi: 10.1101/2020.12.06.413757

[B13] Doudna J. A. Charpentier E. (2014). Genome editing. The new frontier of genome engineering with CRISPR-Cas9. Science 346, 1258096. 10.1126/science.1258096 25430774

[B14] East K. W. Newton J. C. Morzan U. N. Narkhede Y. B. Acharya A. Skeens E. (2020a). Allosteric motions of the CRISPR-Cas9 HNH nuclease probed by NMR and molecular dynamics. J. Am. Chem. Soc. 142, 1348–1358. 10.1021/jacs.9b10521 31885264 PMC7497131

[B15] East K. W. Skeens E. Cui J. Y. Belato H. B. Mitchell B. Hsu R. (2020b). NMR and computational methods for molecular resolution of allosteric pathways in enzyme complexes. Biophys. Rev. 12, 155–174. 10.1007/s12551-019-00609-z 31838649 PMC7040152

[B16] Eggleston A. K. Kowalczykowski S. C. (1991). An overview of homologous pairing and DNA strand exchange proteins. Biochimie 73, 163–176. 10.1016/0300-9084(91)90199-b 1653031

[B17] Gasiunas G. Barrangou R. Horvath P. Siksnys V. (2012). Cas9-crRNA ribonucleoprotein complex mediates specific DNA cleavage for adaptive immunity in bacteria. Proc. Natl. Acad. Sci. U. S. A. 109, E2579–E2586. 10.1073/pnas.1208507109 22949671 PMC3465414

[B18] Gong S. Yu H. H. Johnson K. A. Taylor D. W. (2018). DNA unwinding is the primary determinant of CRISPR-Cas9 activity. Cell Rep. 22, 359–371. 10.1016/j.celrep.2017.12.041 29320733 PMC11151164

[B19] Gorecka K. M. Komorowska W. Nowotny M. (2013). Crystal structure of RuvC resolvase in complex with Holliday junction substrate. Nucleic Acids Res. 41, 9945–9955. 10.1093/nar/gkt769 23980027 PMC3834835

[B20] Hirano S. Nishimasu H. Ishitani R. Nureki O. (2016). Structural basis for the altered PAM specificities of engineered CRISPR-Cas9. Mol. Cell 61, 886–894. 10.1016/j.molcel.2016.02.018 26990991

[B21] Huai C. Li G. Yao R. Zhang Y. Cao M. Kong L. (2017). Structural insights into DNA cleavage activation of CRISPR-Cas9 system. Nat. Commun. 8, 1375. 10.1038/s41467-017-01496-2 29123204 PMC5680257

[B22] Jahagirdar D. Jha V. Basu K. Gomez-Blanco J. Vargas J. Ortega J. (2020). Alternative conformations and motions adopted by 30S ribosomal subunits visualized by cryo-electron microscopy. RNA 26, 2017–2030. 10.1261/rna.075846.120 32989043 PMC7668263

[B23] Jiang F. Doudna J. A. (2017). CRISPR-Cas9 structures and mechanisms. Annu. Rev. Biophys. 46, 505–529. 10.1146/annurev-biophys-062215-010822 28375731

[B24] Jiang F. Taylor D. W. Chen J. S. Kornfeld J. E. Zhou K. Thompson A. J. (2016). Structures of a CRISPR-Cas9 R-loop complex primed for DNA cleavage. Science 351, 867–871. 10.1126/science.aad8282 26841432 PMC5111852

[B25] Jiang F. Zhou K. Ma L. Gressel S. Doudna J. A. (2015). STRUCTURAL BIOLOGY. A Cas9-guide RNA complex preorganized for target DNA recognition. Science 348, 1477–1481. 10.1126/science.aab1452 26113724

[B26] Jinek M. Jiang F. Taylor D. W. Sternberg S. H. Kaya E. Ma E. (2014). Structures of Cas9 endonucleases reveal RNA-mediated conformational activation. Science 343, 1247997. 10.1126/science.1247997 24505130 PMC4184034

[B27] Kowalczykowski S. C. (1991). Biochemistry of genetic recombination: Energetics and mechanism of DNA strand exchange. Annu. Rev. Biophys. Biophys. Chem. 20, 539–575. 10.1146/annurev.bb.20.060191.002543 1831022

[B28] Kraut J. (1988). How do enzymes work. Science 242, 533–540. 10.1126/science.3051385 3051385

[B29] Kunkel T. A. Bebenek K. (2000). DNA replication fidelity. Annu. Rev. Biochem. 69, 497–529. 10.1146/annurev.biochem.69.1.497 10966467

[B30] Leonarski F. D'Ascenzo L. Auffinger P. (2017). Mg^2+^ ions: Do they bind to nucleobase nitrogens? Nucleic Acids Res. 45, 987–1004. 10.1093/nar/gkw1175 27923930 PMC5314772

[B31] Li W. Kamtekar S. Xiong Y. Sarkis G. J. Grindley N. D. Steitz T. A. (2005). Structure of a synaptic gammadelta resolvase tetramer covalently linked to two cleaved DNAs. Science 309, 1210–1215. 10.1126/science.1112064 15994378

[B32] Mitchell B. P. Hsu R. V. Medrano M. A. Zewde N. T. Narkhede Y. B. Palermo G. (2020). Spontaneous embedding of DNA mismatches within the RNA:DNA hybrid of CRISPR-Cas9. Front. Mol. Biosci. 7, 39. 10.3389/fmolb.2020.00039 32258048 PMC7093078

[B33] Nerli S. De Paula V. S. McShan A. C. Sgourakis N. G. (2021). Backbone-independent NMR resonance assignments of methyl probes in large proteins. Nat. Comm. 12, 691. 10.1038/s41467-021-20984-0 PMC7846771 33514730

[B34] Newton M. D. Taylor B. J. Driessen R. P. C. Roos L. Cvetesic N. Allyjaun S. (2019). DNA stretching induces Cas9 off-target activity. Nat. Struct. Mol. Biol. 26, 185–192. 10.1038/s41594-019-0188-z 30804513 PMC7613072

[B35] Nidhi S. Anand U. Oleksak P. Tripathi P. Lal J. A. Thomas G. (2021). Novel CRISPR-Cas Systems: An updated review of the current achievements, applications, and future research perspectives. Int. J. Mol. Sci. 22, 3327. 10.3390/ijms22073327 33805113 PMC8036902

[B36] Nierzwicki L. East K. W. Binz J. Hsu R. V. Ahsan M. Arantes P. R. (2022). Principles of target DNA cleavage and role of Mg^2+^ in the catalysis of CRISPR-Cas9. Nat. Catal. 5, 912–922. 10.1038/s41929-022-00848-6 PMC9909973 36778082

[B37] Nierzwicki L. East K. W. Morzan U. N. Arantes P. R. Batista V. S. Lisi G. P. (2021). Enhanced specificity mutations perturb allosteric signaling in CRISPR-Cas9. Elife 10, e73601. 10.7554/eLife.73601 34908530 PMC8741213

[B38] Nierzwicki L. Palermo G. (2021). Molecular dynamics to predict cryo-EM: Capturing transitions and short-lived conformational states of biomolecules. Front. Mol. Biosci. 8, 641208. 10.3389/fmolb.2021.641208 33884260 PMC8053777

[B39] Nishimasu H. Ran F. A. Hsu P. D. Konermann S. Shehata S. I. Dohmae N. (2014). Crystal structure of Cas9 in complex with guide RNA and target DNA. Cell 156, 935–949. 10.1016/j.cell.2014.02.001 24529477 PMC4139937

[B40] Pacesa M. Lin C. H. Clery A. Saha A. Arantes P. R. Bargsten K. (2021). Structural basis for Cas9 off-target activity. BioRxiv. 10.1016/j.cell.2022.09.026 PMC10103147 36306733

[B41] Pacesa M. Loeff L. Querques I. Muckenfuss L. M. Sawicka M. Jinek M. (2022). R-loop formation and conformational activation mechanisms of Cas9. Nature 609, 191–196. 10.1038/s41586-022-05114-0 36002571 PMC9433323

[B42] Palermo G. Chen J. S. Ricci C. G. Rivalta I. Jinek M. Batista V. S. (2018). Key role of the REC lobe during CRISPR-Cas9 activation by 'sensing', 'regulating', and 'locking' the catalytic HNH domain. Q. Rev. Biophys. 51 (1-11), e91. 10.1017/S0033583518000070 30555184 PMC6292676

[B43] Palermo G. Miao Y. Walker R. C. Jinek M. McCammon J. A. (2017a). CRISPR-Cas9 conformational activation as elucidated from enhanced molecular simulations. Proc. Natl. Acad. Sci. U. S. A. 114, 7260–7265. 10.1073/pnas.1707645114 28652374 PMC5514767

[B44] Palermo G. Ricci C. G. Fernando A. Basak R. Jinek M. Rivalta I. (2017b). Protospacer adjacent motif-induced allostery activates CRISPR-Cas9. J. Am. Chem. Soc. 139, 16028–16031. 10.1021/jacs.7b05313 28764328 PMC5905990

[B45] Palermo G. (2019). Structure and dynamics of the CRISPR-Cas9 catalytic complex. J. Chem. Inf. Model 59, 2394–2406. 10.1021/acs.jcim.8b00988 30763088

[B46] Patel A. C. Palermo G. (2022). Emerging methods and applications to decrypt allostery in proteins and nucleic acids. J. Mol. Biol. 434, 167518. 10.1016/j.jmb.2022.167518 35240127 PMC9398933

[B47] Poitevin F. Kushner A. Li X. Dao Duc K. (2020). Structural heterogeneities of the ribosome: New frontiers and opportunities for Cryo-EM. Molecules 25, 4262. 10.3390/molecules25184262 32957592 PMC7570653

[B48] Raper A. T. Stephenson A. A. Suo Z. (2018). Functional insights revealed by the kinetic mechanism of CRISPR/Cas9. J. Am. Chem. Soc. 140, 2971–2984. 10.1021/jacs.7b13047 29442507

[B49] Ray A. Di Felice R. (2020). Protein-mutation-induced conformational changes of the DNA and nuclease domain in CRISPR/Cas9 systems by molecular dynamics simulations. J. Phys. Chem. B 124, 2168–2179. 10.1021/acs.jpcb.9b07722 32079396

[B50] Ricci C. G. Chen J. S. Miao Y. Jinek M. Doudna J. A. McCammon J. A. (2019). Deciphering off-target effects in CRISPR-Cas9 through accelerated molecular dynamics. ACS Cent. Sci. 5, 651–662. 10.1021/acscentsci.9b00020 31041385 PMC6487449

[B51] Saha A. Ashan M. Arantes P. R. Schmitz M. Chanez C. Jinek M. (2022). An alpha-helical lid guides the target DNA toward catalysis in CRISPR-Cas12a. BioRxiv. 10.1101/2022.09.05.506663 PMC10874386 38368461

[B52] Schmid-Burgk J. L. Gao L. Li D. Gardner Z. Strecker J. Lash B. (2020). Highly parallel profiling of Cas9 variant specificity. Mol. Cell 78, 794–800. 10.1016/j.molcel.2020.02.023 32187529 PMC7370240

[B53] Singh D. Sternberg S. H. Fei J. Doudna J. A. Ha T. (2016). Real-time observation of DNA recognition and rejection by the RNA-guided endonuclease Cas9. Nat. Commun. 7, 12778. 10.1038/ncomms12778 27624851 PMC5027287

[B54] Singh D. Wang Y. Mallon J. Yang O. Fei J. Poddar A. (2018). Mechanisms of improved specificity of engineered Cas9s revealed by single-molecule FRET analysis. Nat. Struct. Mol. Biol. 25, 347–354. 10.1038/s41594-018-0051-7 29622787 PMC6195204

[B55] Slaymaker I. M. Gao L. Zetsche B. Scott D. A. Yan W. X. Zhang F. (2016). Rationally engineered Cas9 nucleases with improved specificity. Science 351, 84–88. 10.1126/science.aad5227 26628643 PMC4714946

[B56] Sternberg S. H. LaFrance B. Kaplan M. Doudna J. A. (2015). Conformational control of DNA target cleavage by CRISPR-Cas9. Nature 527, 110–113. 10.1038/nature15544 26524520 PMC4859810

[B57] Tang H. Yuan H. Du W. Li G. Xue D. Huang Q. (2021). Active-site models of *Streptococcus pyogenes* Cas9 in DNA cleavage state. Front. Mol. Biosci. 8, 653262. 10.3389/fmolb.2021.653262 33987202 PMC8112549

[B58] Wang H. La Russa M. Qi L. S. (2016). CRISPR/Cas9 in genome editing and beyond. Annu. Rev. Biochem. 85, 227–264. 10.1146/annurev-biochem-060815-014607 27145843 PMC13384723

[B59] Wang J. Konigsberg W. H. (2022). Two-metal-ion catalysis: Inhibition of DNA polymerase activity by a third divalent metal ion. Front. Mol. Biosci. 9, 824794. 10.3389/fmolb.2022.824794 35300112 PMC8921852

[B60] Wang J. Natchiar S. K. Moore P. B. Klaholz B. P. (2021a). Identification of Mg^2+^ ions next to nucleotides in cryo-EM maps using electrostatic potential maps. Acta Crystallogr. D. Struct. Biol. 77, 534–539. 10.1107/S2059798321001893 33825713 PMC8025889

[B61] Wang J. Shi Y. Reiss K. Allen B. Maschietto F. Lolis E. (2022a). Insights into binding of single-stranded viral RNA template to the replication-transcription complex of SARS-CoV-2 for the priming reaction from molecular dynamics simulations. Biochemistry 61, 424–432. 10.1021/acs.biochem.1c00755 35199520

[B62] Wang J. Shi Y. Reiss K. Maschietto F. Lolis E. Konigsberg W. H. (2022b). Structural insights into binding of remdesivir triphosphate within the replication-transcription complex of SARS-CoV-2. Biochemistry 61, 1966–1973. 10.1021/acs.biochem.2c00341 36044776 PMC9469760

[B63] Wang J. Skeens E. Arantes P. R. Maschietto F. Allen B. Kyro G. W. (2022c). Structural basis for reduced dynamics of three engineered HNH endonuclease Lys-to-Ala mutants for the clustered regularly interspaced short palindromic repeat (CRISPR)-associated 9 (CRISPR/Cas9) enzyme. Biochemistry 61, 785–794. 10.1021/acs.biochem.2c00127 35420793 PMC9069930

[B64] Wang Y. Mallon J. Wang H. Singh D. Hyun Jo M. Hua B. (2021b). Real-time observation of Cas9 postcatalytic domain motions. Proc. Natl. Acad. Sci. U. S. A. 118, e2010650118. 10.1073/pnas.2010650118 33443184 PMC7812825

[B65] Wodak S. J. Paci E. Dokholyan N. V. Berezovsky I. N. Horovitz A. Li J. (2019). Allostery in its many disguises: From theory to applications. Structure 27, 566–578. 10.1016/j.str.2019.01.003 30744993 PMC6688844

[B66] Xia S. Konigsberg W. H. (2014). RB69 DNA polymerase structure, kinetics, and fidelity. Biochemistry 53, 2752–2767. 10.1021/bi4014215 24720884 PMC4018061

[B67] Xia S. Wang J. Konigsberg W. H. (2013). DNA mismatch synthesis complexes provide insights into base selectivity of a B family DNA polymerase. J. Am. Chem. Soc. 135, 193–202. 10.1021/ja3079048 23214497 PMC3760218

[B68] Zhao L. N. Mondal D. Warshel A. (2020). Exploring alternative catalytic mechanisms of the Cas9 HNH domain. Proteins 88, 260–264. 10.1002/prot.25796 31390092 PMC6942198

[B69] Zhu X. Clarke R. Puppala A. K. Chittori S. Merk A. Merrill B. J. (2019). Cryo-EM structures reveal coordinated domain motions that govern DNA cleavage by Cas9. Nat. Struct. Mol. Biol. 26, 679–685. 10.1038/s41594-019-0258-2 31285607 PMC6842131

[B70] Zuo Z. Liu J. (2017). Structure and dynamics of Cas9 HNH domain catalytic state. Sci. Rep. 7, 17271. 10.1038/s41598-017-17578-6 29222528 PMC5722908

[B71] Zuo Z. Zolekar A. Babu K. Lin V. J. Hayatshahi H. S. Rajan R. (2019). Structural and functional insights into the bona fide catalytic state of *Streptococcus pyogenes* Cas9 HNH nuclease domain. Elife 8, e46500. 10.7554/eLife.46500 31361218 PMC6706240

